# Cigarette smoking abstinence at follow-up at 12 months among US adults who regularly used Electronic Nicotine Delivery Systems and smoked in the past year: A prospective cohort study

**DOI:** 10.18332/tid/215874

**Published:** 2026-01-22

**Authors:** Amy L. Nyman, Katherine C. Henderson, David L. Ashley, Claire A. Spears, Jidong Huang, Zongshuan Duan, Scott R. Weaver

**Affiliations:** 1 Department of Health Policy and Behavioral Sciences, School of Public Health, Georgia State University, Atlanta, United States; 2 Department of Population Health Sciences, School of Public Health, Georgia State University, Atlanta, United States; 3 TRS Consulting56 LLC, Lilburn, Georgia, United States

**Keywords:** electronic nicotine delivery systems (ENDS), Juul, Vuse Alto, cigarette smoking, cessation

## Abstract

**INTRODUCTION:**

Regular use of electronic nicotine delivery systems (ENDS) by people who smoke cigarettes may impact smoking trajectories. ENDS brands are used by different populations in different ways, but their associations with smoking cessation are not well understood. This study evaluated whether regular use of Juul or Alto ENDS differently impacted smoking abstinence one year later among adults who had smoked cigarettes.

**METHODS:**

This prospective cohort study surveyed a national sample of US adults who used ENDS in 2022–2023 and again after one year to assess cigarette smoking. Multivariable logistic regression models used data from 237 people who had smoked cigarettes in the past year and regularly used ENDS products Juul or Alto at baseline to examine the characteristics and behaviors associated with abstaining from cigarette smoking at follow-up at 12 months.

**RESULTS:**

Whereas no overall differences in smoking abstinence at follow-up at 12 months were found between adults who used Juul versus adults who used Alto, adults who used Juul and had quit smoking by baseline were more likely than their Alto-using counterparts to remain abstinent at follow-up at 12 months (AOR=7.07). Other characteristics that were associated with abstaining from cigarettes at follow-up included being 18–29 years (vs older) (AOR=3.64), identifying as White, non-Hispanic (vs another race/ethnicity) (AOR=3.03), not currently smoking at baseline (vs currently smoking) (AOR=20.25), using their Juul or Alto product to quit smoking or remain quit (AOR=2.77), and use of menthol cigarette flavors (vs tobacco flavor) (AOR=2.54).

**CONCLUSIONS:**

This longitudinal study found limited differences in smoking abstinence after one year between those who regularly used Juul versus Alto. However, people who used ENDS products specifically to quit smoking were more likely to achieve smoking abstinence and there were important sociodemographic differences. Future research is needed to inform interventions to increase the likelihood that people who use ENDS completely stop smoking and eventually quit all consumer nicotine products.

## INTRODUCTION

There is much debate over the impact of using electronic nicotine delivery systems (ENDS) on cigarette smoking trajectories, with numerous studies concluding ENDS use may aid smoking cessation^1,2^ and others suggesting ENDS use may sustain dual use or inhibit quitting^3,4^. Understanding of the long-term outcomes of ENDS use on cigarette smoking can ensure future scientific and policy recommendations that are in the best interest of public health.

Many studies examining the impact of ENDS use on smoking cessation focus on ENDS use in general^5^ or on one specific ENDS product^6^, and therefore do not compare the impact of similar ENDS models from different brands. However, some recent research highlights differences in user characteristics, reasons for use, or usage patterns by ENDS brand or model^7-9^, indicating a need to understand the impact of use of comparable products on subsequent cigarette smoking outcomes. If one product is more effective at promoting smoking cessation and its users differ sociodemographically, some populations may be differentially impacted, with implications for health equity. A recent study found Juul and Puff Bar were more often reported as the usual brand for Black and Hispanic youth and young adults than by those of other races and ethnicities^8^. Another study noted ENDS device type differences between female and male ENDS users^9^. Still other recent findings indicate those who have smoked cigarettes in the past year who regularly use Juul were less likely to not be currently smoking, were younger, and more likely to identify as a racial or ethnic minoritized group than those who regularly use Vuse Alto^7^. Longitudinal studies following users of similar ENDS products who currently or recently smoked cigarettes are necessary to understand the ultimate effects of dual use on cessation.

This study examines differences in sociodemographic characteristics and usage patterns of leading ENDS products Juul and Alto, and measures cigarette smoking abstinence after one year among those who currently or recently smoked cigarettes at baseline. We aim to determine whether regular usage of two comparable ENDS products is associated with differential smoking outcomes at follow-up at 12 months, in interaction with or independent of sociodemographic or behavioral characteristics.

## METHODS

### Participants and procedures

This prospective cohort study included a national sample of 759 US adults who currently or formerly smoked combusted cigarettes, used select market-leading ENDS brands, and participated in a 12-month longitudinal survey study assessing cigarette smoking and ENDS usage (September 2022–July 2024). Recruitment occurred via paid social media advertisements from September 2022 through July 2023. To be eligible for the larger study, participants needed to be at least 18 years old, current US residents, and had to report past 30-day use of one or more specific ENDS products. Participants who completed the baseline survey and had smoked cigarettes in the past year, as well as those who had used an ENDS product that was unauthorized for sale in the US at the time of the baseline survey, were invited to complete the follow-up survey 12 months later. This study focuses on the subsample that smoked cigarettes in the past year and regularly used Juul or Alto (but not both) at the baseline survey. Of the 320 participants who reported past-year cigarette smoking and regular use of either Juul or Alto (but not both) at the baseline survey, the analytic sample for this study uses data from the 237 participants who completed the survey at follow-up at 12 months and were not missing data on the covariates included in the present study (see Supplementary file Figure S1 for a flowchart of participant details). Full study details have been previously published^7^.

### Measures

The outcome was smoking abstinence at follow-up at 12 months. The independent variable was whether participants regularly used Juul versus Alto at baseline. Covariates included nicotine concentration, most commonly used Juul/Alto flavor (tobacco vs menthol/mint), number of days Juul/Alto used in the past month, using Juul or Alto to quit smoking or remain quit, current cigarette smoking status, flavor of respondents’ regular cigarette brand (tobacco vs menthol), presence of serious psychological distress, and sociodemographic characteristics (age, gender, race/ethnicity, education level, sexual orientation) at baseline, details of which are given in Supplementary file Table S1 and described elsewhere^7^.

### Data analysis

A main-effects multivariable logistic regression model was used to predict smoking abstinence at follow-up at 12 months, with adjustment for covariates. Subsequent models examined two-way interactions between Juul versus Alto use and each covariate, and the three-way interaction with cigarette flavor and Juul or Alto flavor. Simple main effects were examined where a significant interaction effect was obtained. Odds ratios (ORs) with 95% profile likelihood confidence intervals were obtained with SPSS v. 29 ^10^.

## RESULTS

Nearly two-thirds of the sample (62.4%) were smoking abstinent at follow-up at 12 months, including 40.0% of people who currently smoked at baseline. Adjusting for all covariates, there was no difference in the odds of smoking abstinence between Juul versus Alto users (Table 1, Model 1). However, interaction analyses indicated that this association differed according to age and baseline smoking status. Juul use was positively, albeit non-significantly associated (vs Alto use) with smoking abstinence among those aged ≥30 years (AOR=1.34), whereas it was negatively, albeit also non-significantly associated with smoking abstinence among young adults (aged 18–29 years) (AOR=0.20) (Model 2b). Among those who were currently smoking cigarettes at baseline, there was no significant difference in smoking abstinence between Juul and Alto users; whereas among those who were past year but not currently smoking at baseline, Juul users were more likely than Alto users to have remained abstinent at follow-up at 12 months (AOR=7.07) (Model 3b). No other interaction effects were statistically significant (Models 4–12).

**Table 1:** Predictors of smoking abstinence at follow-up ^a^ at 12 months among US adults who smoked within the past year and regularly used either Juul or Vuse Alto ^b^ at baseline, results from a prospective cohort study, 2022–2024 (N=237)

*Variables*	*% (n)*	*AOR ^c^*	*95% CI*
		*Model 1: Main effects model*	
**Regular ENDS product**			
Juul	42.6 (101)	0.94	0.45–1.98
Alto ®	57.4 (136)	1	
**Age** (years)			
18–29	21.5 (51)	**3.64^d^**	**1.48–9.66**
≥30 ®	78.5 (186)	1	
**Gender**			
Cis Male	35.4 (84)	1.05	0.52–2.13
Cis Female ®	64.6 (153)	1	
**Race/ethnicity**			
White, NH	74.3 (176)	**3.03**	**1.38–6.90**
Other ®	25.7 (61)	1	
**Education level**			
Lower than Bachelor’s degree	73.4 (174)	0.93	0.42–2.04
Bachelor’s degree or higher ®	26.6 (63)	1	
**Sexual orientation**			
Not a sexual minority	75.5 (179)	1.00	0.44–2.30
Sexual minority ®	24.5 (58)	1	
**Serious psychological distress (SPD)** ^e^			
No SPD	77.2 (183)	0.92	0.40–2.10
SPD ®	22.8 (54)	1	
**Baseline cigarette smoking status**			
Not currently smoking	43.0 (102)	**20.25**	**8.99–51.75**
Currently smoking ®	57.0 (135)	1	
**Nicotine content of [Juul/Alto]**			
≤3%	23.6 (56)	1.03	0.43–2.48
5% ®	76.4 (181)	1	
**Flavor of [Juul/Alto] used most often**			
Menthol/Mint	60.3 (143)	1.04	0.40–2.72
Tobacco ®	39.7 (94)	1	
**Days used [Juul/Alto] per month**			
15–25	16.9 (40)	1.30	0.53–3.22
26–30 ®	83.1 (197)	1	
**Using [Juul/Alto] to quit cigarettes/remain quit**			
Using to quit/remain quit	77.2 (183)	**2.77**	**1.19–6.72**
Not using to quit/remain quit ®	22.8 (54)	1	
**Baseline regular flavor of cigarettes**			
Menthol	51.5 (122)	**2.54**	**1.02–6.53**
Tobacco ®	48.5 (115)	1	
		***Model 2a: Regular ENDS product by age***	
Juul (vs Alto)		1.34	0.59–3.07
Age (years) 18–29 (vs ≥30)		**10.75**	**2.64–59.97**
Regular ENDS product × age		**0.15**	**0.02––0.90**
		***Model 2b: Regular ENDS product at age 18–29 years***	
Juul (vs Alto)		0.20	0.03–1.04
		***Model 3a: Regular ENDS product by baseline smoking***	
Juul (vs Alto)		0.63	0.27–1.43
Not currently smoking at baseline (vs currently smoking)		**9.66**	**3.76–27.98**
Regular ENDS product × baseline smoking status		**11.26**	**1.47–240.86**
		***Model 3b: Regular ENDS product at not currently smoking at baseline***	
Juul (vs Alto)		**7.07**	**1.08–141.21**
		***Model 4: Regular ENDS product by gender***	
Juul (vs Alto)		0.78	0.31–1.97
Male (vs Female)		0.83	0.30–2.25
Regular ENDS product × gender		1.60	0.39–6.63
		***Model 5: Regular ENDS product by race***	
Juul (vs Alto)		0.76	0.18–3.11
White, NH (vs Other)		2.64	0.88–8.21
Regular ENDS product × race/ethnicity		1.35	0.26–7.21
		***Model 6: Regular ENDS product by education level***	
Juul (vs Alto)		1.68	0.41–6.89
Lower than Bachelor’s degree (vs Bachelor’s degree or higher)		1.46	0.42–5.01
Regular ENDS product × education level		0.46	0.09–2.29
		***Model 7: Regular ENDS product by sexual orientation***	
Juul (vs Alto)		0.39	0.08–1.86
Not a sexual minority (vs sexual minority)		0.69	0.24–1.91
Regular ENDS product × sexual orientation		3.03	0.53–19.02
		***Model 8: Regular ENDS product by SPD (Ref: Alto)***	
Juul (vs Alto)		0.50	0.11–2.23
No SPD (vs SPD)		0.62	0.19–1.97
Regular ENDS product × serious psychological distress (SPD)		2.25	0.43–12.58
		***Model 9: Regular ENDS product by ENDS nicotine concentration***	
Juul (vs Alto)		0.97	0.43–2.23
≤3% nicotine (vs 5% nicotine)		1.09	0.37–3.27
Regular ENDS product × nicotine content of [Juul/Alto]		0.86	0.16–4.84
		***Model 10: Regular ENDS product by ENDS flavor by cigarette flavor***	
Juul (vs Alto)		1.45	0.45–4.79
ENDS flavor menthol/mint (vs tobacco)		1.1	0.24–5.10
Cigarette flavor menthol (vs tobacco)		3.37	0.36–32.25
Regular ENDS product × flavor of [Juul/Alto] used most often		1.16	0.13–10.11
Regular ENDS product × cigarette flavor used most often		0.70	0.01–37.82
Flavor of [Juul/Alto] used most often × cigarette flavor used most often		1.19	0.09–16.83
Regular ENDS product × flavor of [Juul/Alto] used most often × cigarette flavor used most often		0.45	0.01–37.93
		***Model 11: Regular ENDS product by days per month used***	
Juul (vs Alto)		0.98	0.44–2.22
15–25 days used per month (vs 26–30 days used per month)		1.46	0.39–5.65
Regular ENDS product × days used [Juul/Alto] per month		0.81	0.13–4.80
		***Model 12: Regular ENDS product by use of product to quit smoking***	
Juul (vs Alto)		1.41	0.31–6.53
Using product to quit (vs not using product to quit)		**3.65**	**1.06–13.50**
Regular ENDS product × using [Juul/Alto] to quit cigarettes		0.60	0.11–3.25

AOR: adjusted odds ratio.

® Reference categories. NH: non-Hispanic. ENDS: Electronic Nicotine Delivery Systems. More detail on question wording and coding can be found in Supplementary file Table S1.

a A response of ‘not at all’ to the question ‘Do you now smoke cigarettes every day some days, or not at all?’ at follow-up at 12 months was considered smoking abstinence.

b Regular use of Juul or Vuse Alto is defined as using the product ≥15 days in the past month and ≥1 pods in an average week and not using the other product >4 days in the past month or ≥1 pods in an average week.

c Generalized linear models were used to obtain odds ratios (AOR) adjusted for all covariates shown to predict likelihood of quitting cigarette smoking or remaining quit at follow-up at 12 months. Models 2–12 examined interaction effects and included all covariates, not shown for brevity, in Model 1. Where a significant interaction effect was obtained, the analysis was re-run with the dummy coding of the interacting variable flipped to obtain the conditional effect of Juul vs Alto at the level of the moderating variable (Models 1b and 2b).

d Bold values indicate statistical significance based on 95% CI for the AOR not overlapping with 1.0.

e Kessler-6 Distress scale: scores >12 indicate a high probability of serious mental illness with significant impairment.

Younger adults (aged 18–29 years) (AOR=3.64), those identifying as non-Hispanic White (AOR=3.03), not currently smoking at baseline (AOR=20.25), using their product (Juul or Alto) to quit smoking (AOR=2.77), or smoking menthol cigarettes (AOR=2.54) were each more likely to be smoking abstinent at follow-up at 12 months than their respective counterparts, adjusting for all other covariates (Table 1, Model 1). No significant differences in smoking abstinence were observed for gender, education level, sexual orientation, serious psychological distress, nicotine concentration, Juul/ Alto flavor, or days used per month when adjusting for other covariates.

## DISCUSSION

In July 2025, the US FDA authorized marketing of Juul, which joined Alto and a limited list of other ENDS products as authorized for sale in the US^11^, after retracting a prior market denial order for Juul^12^. Understanding differences in cigarette smoking outcomes between Juul and Alto, two US market-leading brands, could inform consumers, health researchers and practitioners, and regulators. This novel study compares smoking outcomes between adults who used Juul versus Alto using longitudinal data.

Prior cross-sectional research found differences in readiness to quit, use of ENDS to quit smoking, and sociodemographic characteristics between those who use Alto versus Juul. The study reported that those who smoke cigarettes and use Alto might have greater success in subsequently quitting smoking^7^. Moreover, while Juul and Alto are both similarly designed closed-system pod ENDS with similar levels of nicotine concentration in a nicotine-salt formulation, Alto may generate greater nicotine yields^13^. However, the current prospective cohort study found no overall differences in smoking abstinence at follow-up at 12 months between those who regularly used Juul and those who regularly used Alto at baseline. Yet among those who smoked in the past year but not currently at baseline, participants were more likely to be abstinent 12 months later if they regularly used Juul than if they regularly used Alto. This suggests that those who formerly smoked who use Juul are less likely to subsequently relapse to smoking than former smokers who use Alto. However, our results indicate tentative evidence that this pattern might not extend to younger adults. Specifically, we found younger adults who regularly used Juul might be at a relative disadvantage compared to those who used Alto based on an age by Juul versus Alto use interaction. Despite this interaction, the statistical effect for Juul versus Alto was non-significant among both younger adults and adults aged >29 years, suggesting that this study may be insufficiently powered to detect a difference for one or both age groups. Further research with larger samples will be needed to reconcile these findings.

While randomized controlled trials have supported the efficacy of newer generation ENDS for smoking cessation, longitudinal observational studies have been mixed^1,14^. More than three-fourths of our sample reported they were using Juul or Alto to abstain from smoking at baseline, and 62.4% were self-reported abstinent from smoking at follow-up. However, this study was not designed to evaluate the efficacy or effectiveness of Juul or Alto for smoking cessation, and generalization of findings is limited by the convenience sample and lack of biochemical verification of smoking abstinence.

Interestingly, this study found those who smoked menthol cigarettes were more likely to be abstinent at follow-up than those who smoked non-menthol cigarettes. Whereas this is in contrast to a prior research finding that adults who smoke menthol cigarettes were less likely to quit smoking, the prior study also found that ENDS use was associated with smoking cessation and more strongly for those who smoked menthol cigarettes^15^. Furthermore, our study adjusted for ethnic/racial minoritized status, which is associated with both menthol cigarette use and poor smoking cessation outcomes.

### Limitations

This study has several limitations. First, use of a convenience sample limits the generalizability of the findings. Second, this study relies on observational data at two time points with a one-year interval, thus limiting causal inference and examination of detailed trajectories of product use and smoking behavior. Finally, self-reports of product use may lead to misclassification and social desirability bias.

## CONCLUSIONS

Though differences exist in the characteristics and usage patterns of those who regularly use Juul and Alto ENDS products, this prospective cohort study found very limited differences in smoking abstinence after one year among those who had currently or recently smoked cigarettes at baseline and regularly used either Juul or Alto. Relapse to smoking may be less likely with Juul than with Alto, which warrants further study.

## Supplementary Material



## Data Availability

The data supporting this research are available from the following source: https://doi.org/10.57709/693T-TY29
