# Supplementary material for: Cigarette smoking abstinence at follow-up at 12 months among US adults who regularly used Electronic Nicotine Delivery Systems and smoked in the past year: A prospective cohort study
Source: Tob Induc Dis. 2026 Jan 22;24:10.18332/tid/215874. doi: 10.18332/tid/215874 (PMC12825412; doi:10.18332/tid/215874)
Supplement: Supplementary file 1 [file TID-24-08-s1.pdf]

Figure S1. Participant flowchart from completed baseline surveys through follow-up analytic sample: U.S. adults 2022-2024

[Description: Flowchart depicting the numbers of participants involved in each step between completion of baseline survey and final follow-up analytic sample.]

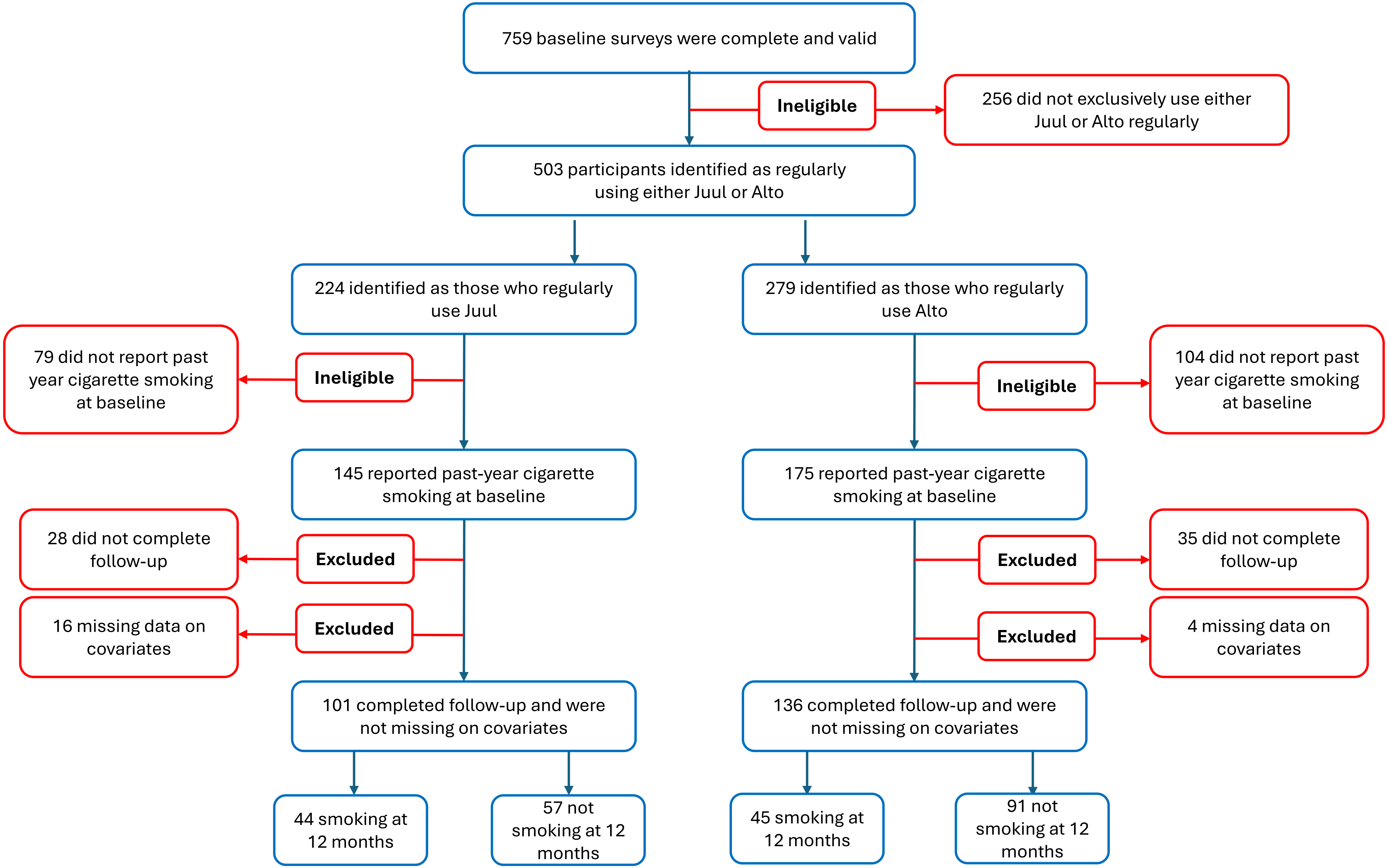
759 baseline surveys were complete and valid
Ineligible
256 did not exclusively use either Juul or Alto regularly
503 participants identified as regularly using either Juul or Alto
224 identified as those who regularly use Juul
279 identified as those who regularly use Alto
Ineligible
79 did not report past year cigarette smoking at baseline
Ineligible
104 did not report past year cigarette smoking at baseline
145 reported past-year cigarette smoking at baseline
175 reported past-year cigarette smoking at baseline
Excluded
28 did not complete follow-up
Excluded
16 missing data on covariates
Excluded
35 did not complete follow-up
Excluded
4 missing data on covariates
101 completed follow-up and were not missing on covariates
136 completed follow-up and were not missing on covariates
44 smoking at 12 months
57 not smoking at 12 months
45 smoking at 12 months
91 not smoking at 12 months

Table S1. Detailed Description of Measures and Variable Construction

Construct	Question Text	Response Options	Operationalization
Outcome variable (Follow-up)			
Smoking abstinence at 12-month follow-up	(1) Do you now smoke cigarettes…	0=Not at all<br>1=Some days<br>2=Every day	If (1) = 0, then abstinent<br>If (1) > 0, then smoking
Predictor Variables (Baseline)			
Regular use of Juul or Alto	(1) On how many of the past 30 days did you use the following product(s)? [Juul]<br><br>(2) On how many of the past 30 days did you use the following product(s)? [Alto]<br><br>(3) On average, how many of the following product(s) do you use in a week? [Juul pods]<br><br>(4) On average, how many of the following product(s) do you use in a week? [Alto pods]	(1) 0 – 30<br><br>(2) 0 – 30<br><br>(3) # used<br><br>(4) # used	If (1) > 14 and (3) >= 1 and [(2) < 5 or (4) < 1], then regular use of Juul<br><br>Or<br><br>If (2) > 14 and (4) >= 1 and [(1) < 5 or (3) < 1], then regular use of Alto
Age	(1) What is your birthdate?<br><br>[If (1) is "Don't know", "Refused" or blank]<br>(2) About how old are you?	(1) MM/DD/YYYY<br><br>(2) Years	Variable created from (1) to age in years
Gender	(1) What terms best express how you describe your gender identity? Please select one or more.	1 = Man/Male<br>2 = Woman/Female<br>3 = Non-binary<br>4 = Transgender	If (1) = 1 and not 2-7, Cisgender male<br>If (1) = 2 and not 1 or 3-7, Cisgender female<br>If (1) = 8, NA

		5 = Genderqueer, Gender Nonconforming, or Genderfluid<br>6 = Agender<br>7 = None of these describe me, and I want to specify	If (1) = else, Other
Race/Ethnicity	(1) What race or races do you consider yourself to be? Please select one or more.	(1) 1 = American Indian or Alaska Native<br>2 = Asian<br>3 = Black or African American<br>4 = Native Hawaiian or Pacific Islander<br>5 = White<br>6 = Other<br>7 = Don't know	If (2) = 1, Hispanic<br><br>If (1) = only 5 and (2) not equal to 1, White non-Hispanic<br><br>If (1) = only 3 and (2) not equal to 1, Black non-Hispanic<br><br>If (1) = else and (2) not equal to 1, Other
	(2) Do you consider yourself to be of Hispanic, Latinx, or of Spanish origin?	(2) 1 = Yes, 0 = No, 9 = Don't know	
Education	(1) What is the highest grade or level of school you have completed or the highest degree you have received?	12 = 12th grade or less, no diploma<br>13 = High school graduate<br>14 = GED or equivalent<br>15 = Some college, no degree<br>16 = Associate degree<br>17 = Bachelor's degree (Example: BA, AB, BS, BBA)<br>18 = Master's degree (Example: MA, MS, MEng, MEd, MBA)<br>19 = Professional school degree (Example: MD, DDS, DVM, JD)<br>20 = Doctoral degree (Example: PhD, EdD)	If (1) < 17, Less than Bachelor's degree<br><br>If (1) > 16, Bachelor's degree plus

Sexual Orientation	(1) Which of the following best represents how you think of yourself?	1 = Gay<br>2 = Lesbian<br>3 = Straight; that is, not gay or lesbian, etc.<br>4 = Bisexual<br>5 = Other	If (1) = 3, Not sexual minoritized<br><br>If (1) = else, Sexual minoritized
Serious Psychological Distress	During the past 30 days, about how often did you feel:<br>(1) Nervous<br>(2) Hopeless<br>(3) Restless or fidgety<br>(4) So depressed that nothing could cheer you up<br>(5) That everything was an effort<br>(6) Worthless	4 = All of the time, 3 = Most of the time, 2 = Some of the time, 1 = A little of the time, 0 = None of the time	If sum of (1), (2), (3), (4), (5), (6) < 13, No serious psychological distress<br><br>If sum of (1), (2), (3), (4), (5), (6) > 12, Serious psychological distress
Baseline Cigarette Smoking Status	(1) Have you ever smoked a cigarette, even one or two puffs?<br><br>(2) How many cigarettes have you smoked in your entire life? A pack usually has 20 cigarettes in it.	(1) 1 = Yes, 0 = No<br><br>(2) 1 = 1 or more puffs but never a whole cigarette<br>2 = 1 to 10 cigarettes (about ½ pack total)<br>3 = 11 to 20 cigarettes (about ½ pack to 1 pack)<br>4 = 21 to 99 cigarettes (more than 1 pack but less than 5 packs)<br>5 = 100 or more cigarettes (5 packs or more)	If (1) = 0 or (2) < 5, never smoked [Not currently smoking]<br><br>If (2) = 5 and (3) = 0, formerly smoked [Not currently smoking]<br><br>If (2) = 5 and (3) = 2, currently smoke every day [Currently smoking]<br><br>If (2) = 5 and (3) = 1, currently smoke some days [Currently smoking]

	(3) Do you now smoke cigarettes…	(3) 2 = Every day, 1 = Some days, 0 = Not at all	
Nicotine Content of Regular Product	(1) What percentage nicotine are the Juul pods you most often use?<br><br>(2) What percentage nicotine are the Altos you most often use?	(1) 1 = 3% nicotine<br>2 = 5% nicotine<br>3 = Other (Please specify)<br>9 = Don't know<br><br>(2) 1 = 1.8% nicotine<br>2 = 2.4% nicotine<br>3 = 5.0% nicotine<br>4 = Other (Please specify)<br>9 = Don't know	If (1) = 1 or if (2) = 1 or 2, 3% or less nicotine<br>If (1) = 2 or (2) = 3, 5% nicotine<br>If (1) = 3 or (2) = 4, Other<br>If (1) = 9 or (2) = 9, Don't Know
Flavor of Regular Product Used Most Often	(1) Which flavor of Juul [do] you use most often?<br><br>(2) Which flavor of Alto [do] you use most often?	(1) 1 = Menthol<br>2 = Virginia Tobacco<br>3 = Some other flavor (Please specify)<br>4 = Other - Mint<br><br>(2) 1 = Menthol<br>2 = Golden Tobacco flavor<br>3 = Rich Tobacco flavor<br>4 = Some other flavor (Please specify)<br>5 = Other – Mint	If (1) = 1 or 4, or (2) = 1 or 5, Menthol/mint<br>If (1) = 2, or (2) = 2 or 3, Tobacco
Days Per Month Used Regular Product	On how many of the past 30 days did you use [regular product]?	0 – 30 days	Unchanged
Using Regular Product to Quit Cigarettes	Are you currently using [regular product] to quit smoking regular cigarettes or to remain quit?	1 = Yes, 0 = No	Unchanged

Flavor of regular brand of cigarettes	Is/was your [regular/last] brand of cigarettes flavored to taste like menthol or mint?	1 = Yes, 0 = No	Unchanged

ENDS = Electronic Nicotine Delivery Systems
# = Number
NA = Not Applicable

© 2026 Nyman A.L. et al.
